# Optimizing conservation planning for multiple cohabiting species

**DOI:** 10.1371/journal.pone.0234968

**Published:** 2020-06-22

**Authors:** Yicheng Wang, Qiaoling Fang, Sahan T. M. Dissanayake, Hayri Önal

**Affiliations:** 1 College of Resources and Environment, Qingdao Agricultural University, Qingdao, China; 2 College of Management, Ocean University of China, Qingdao, China; 3 Department of Economics, Portland State University, Portland, OR, United States of America; 4 Department of Agricultural and Consumer Economics, University of Illinois at Urbana-Champaign, Urbana, IL, United States of America; Institute of Geographic Sciences and Natural Resources Research Chinese Academy of Sciences, CHINA

## Abstract

Conservation planning often involves multiple species occupying large areas including habitat sites with varying characteristics. For a given amount of financial resources, designing a spatially coherent nature reserve system that provides the best possible protection to targeted species is an important ecological and economic problem. In this paper, we address this problem using optimization methods. Incorporating spatial criteria in an optimization framework considering spatial habitat needs of multiple species poses serious challenges because of modeling and computational complexities. We present a novel linear integer programming model to address this issue considering spatial contiguity and compactness of the reserved area. The model uses the concept of path in graph theory to ensure contiguity and minimizes the sum of distances between selected sites and a central site in individual reserves to promote compactness. We test the computational efficiency of the model using randomly generated data sets. The results show that the model can be solved quite efficiently in most cases. We also present an empirical application of the model to simultaneous protection of two cohabiting species, Gopher Tortoise and Gopher Frogs, in a military installation in Georgia, USA.

## Introduction

Conservation nature reserves are the primary means for protecting biological diversity. Such protected areas are established to serve usually multiple (often many) species coexisting in the same area [1–3]. Various techniques have been introduced for designing nature reserves for multiple cohabiting species, including site scoring, gap analysis, and heuristics. In this paper, we address this issue using an optimization framework.

Nature reserves can be ecologically effective only if they possess certain spatial properties [4, 5]. It has long been acknowledged that spatial attributes, such as size, connectivity (or contiguity), compactness, proximity, habitat corridors, and presence of core and buffer zones affect movement behaviors and long-term persistence of species [6]. Depending on particular concerns in each case, one or more of these spatial attributes need to be considered alone or simultaneously. Furthermore, consideration of each attribute may be species-specific because species usually differ in spatial characteristics of their habitat needs. Among the spatial attributes, reserve contiguity has been of particular interest; therefore, it has been studied extensively. Some studies addressed compactness, sometimes in combination with contiguity, to improve the ecological effectiveness of the reserve. In addition to ecological effectiveness, reserve contiguity and compactness may also offer economic benefits such as management convenience and cost effectiveness.

Integer programming has been a widely used modeling approach for determining the optimal conservation reserve design. Initial optimization studies have adopted the basic set covering and maximal covering formulations in the operations research field [7–13]. Following those earlier studies, some integer programming formulations have successfully incorporated spatial attributes in reserve site selection (see [6, 14, 15] for reviews). Several recent papers used networks and graph theoretic concepts to model reserve contiguity [16–22], contiguity and compactness [23–25], and connecting corridors [26–28]. Most of these studies considered only one spatial attribute, or, when multiple attributes were involved, the analysis was restricted to only one species. Most contiguity formulations mentioned above find an entirely contiguous reserve where selected sites are physically connected, but contiguity is not species-specific, namely, the sites dedicated to the protection of a species that requires a fully connected habitat may not necessarily be connected through habitat areas suitable for that species.

Only a few linear integer programming formulations have been presented recently to optimize the design of contiguous and compact reserves with joint consideration of multiple species. The model presented by Marianov et al. [29] promotes contiguity and compactness by assembling candidate sites into 1-, 2- and 4-site possible reserves and selects from those reserves to satisfy the habitat size requirements specified for different species. Their method would be difficult to use when the shapes of candidate sites are irregular and/or a large reserve is needed. The model presented by Wang and Önal [25] ensures contiguity by employing a method used in the sales territory alignment problem [30]. Their model promotes compactness by minimizing the sum of shortest distances between selected sites that make up individual reserves and the centers of those reserves, which are determined simultaneously. Although a practical anomaly has not been reported by Wang and Önal, it is theoretically possible that shortest distance calculations in the model may involve sites that are not selected (i.e., they may lie outside of the reserve).

In this paper, we present a novel 0–1 programming model for optimizing the configuration of contiguous and compact reserves for multiple cohabiting species. Contiguity is enforced by employing graph theoretic concepts, in particular the concept of *path*. Compactness is promoted by minimizing the sum of shortest-path distances between selected sites and the centers of reserves designated to individual species, summed across all species. A similar approach was used previously by Wang and Önal [25]. However, unlike in Wang and Önal, the model we introduce here ensures that the shortest-path distances between selected sites and reserve centers involve only the paths that are comprised of selected reserve sites. The model configures reserves to protect a minimum viable population for each target species with limited availability of conservation resources. The details will be explained in the following section.

## Materials and methods

We follow the conventional approach in the reserve design/site selection literature by dividing the region in which conservation reserves are to be established into sites (parcels or areas) with regular or irregular shapes. We used a square grid for convenience, but our method is applicable to other regular and irregular shapes as well. Two sites are assumed to be adjacent if they share a common edge or corner, and they are connected if they are linked to each other by a chain of mutually adjacent reserved sites. Note that both the adjacency and connectivity properties can be defined on a species basis. For example, for avian species, two sites may be considered ‘adjacent’ if they are within the species’ dispersal capacity (i.e., within a fly-over distance). A reserve designated to a species is contiguous if any two sites in it are connected through a chain of mutually adjacent reserve sites. We assume that the distribution of species across sites, the species-specific habitat quality, and the acquisition cost of each site are known.

We aim to configure a reserve system that includes a specified number of reserves for individual species, where each reserve supports a viable population of the designated species and accommodates its spatial needs. We configure each reserve around a central site, which is selected by the model together with the other sites in that reserve. Each reserve is associated with a *directed planar graph* where the nodes (denoted by *i*) are in a one-to-one correspondence with the sites in that reserve and a directed arc is defined for each pair of adjacent sites. A *path* from node *i*_0_ to *i*_*p*_ is a sequence of arcs *P* = {(*i*_0_,*i*_1_),(*i*_1_,*i*_2_),⋯,(*i*_*p*−1_,*i*_*p*_)} in which the initial node of each arc is the terminal node of the preceding arc in the sequence [31]. We require the node associated with each site to be connected to the node associated with a *central* site in the reserve. This ensures that the reserve is fully connected. For each central site, we define a path that connects it directly to an *auxiliary node O* which, for modeling reasons, we put outside of the conservation area and designate as the sink node. This allows connecting non-central sites to *O* also, namely through the path that connects each non-central site to the central site and an auxiliary arc connecting the central site to *O*. Williams [32] used a similar modeling approach to achieve contiguity in wildlife corridors. His model involves no species consideration, however, and the reserves to be connected exist as a priori. In our problem, multiple species must be handled together and selection of all sites that form the reserves, including the central site in each reserve, are determined simultaneously by the model. We define the distance between a selected site and the central site of the reserve it belongs as the total length of all arcs included in the path that connects the site to the center. We use the overall distance between selected sites and their respective centers across all species as a measure of the compactness of the reserve system.

We require that each selected site belongs to only one reserve designated to a given species. This avoids double-counting when calculating the site’s contribution to the protection of a designated species. A site can belong to different reserves that serve different species, however. This encourages the selection of overlapping reserves to improve the compactness of the reserve system without requiring excessive sites and use of conservation resources.

**Fig 1:**
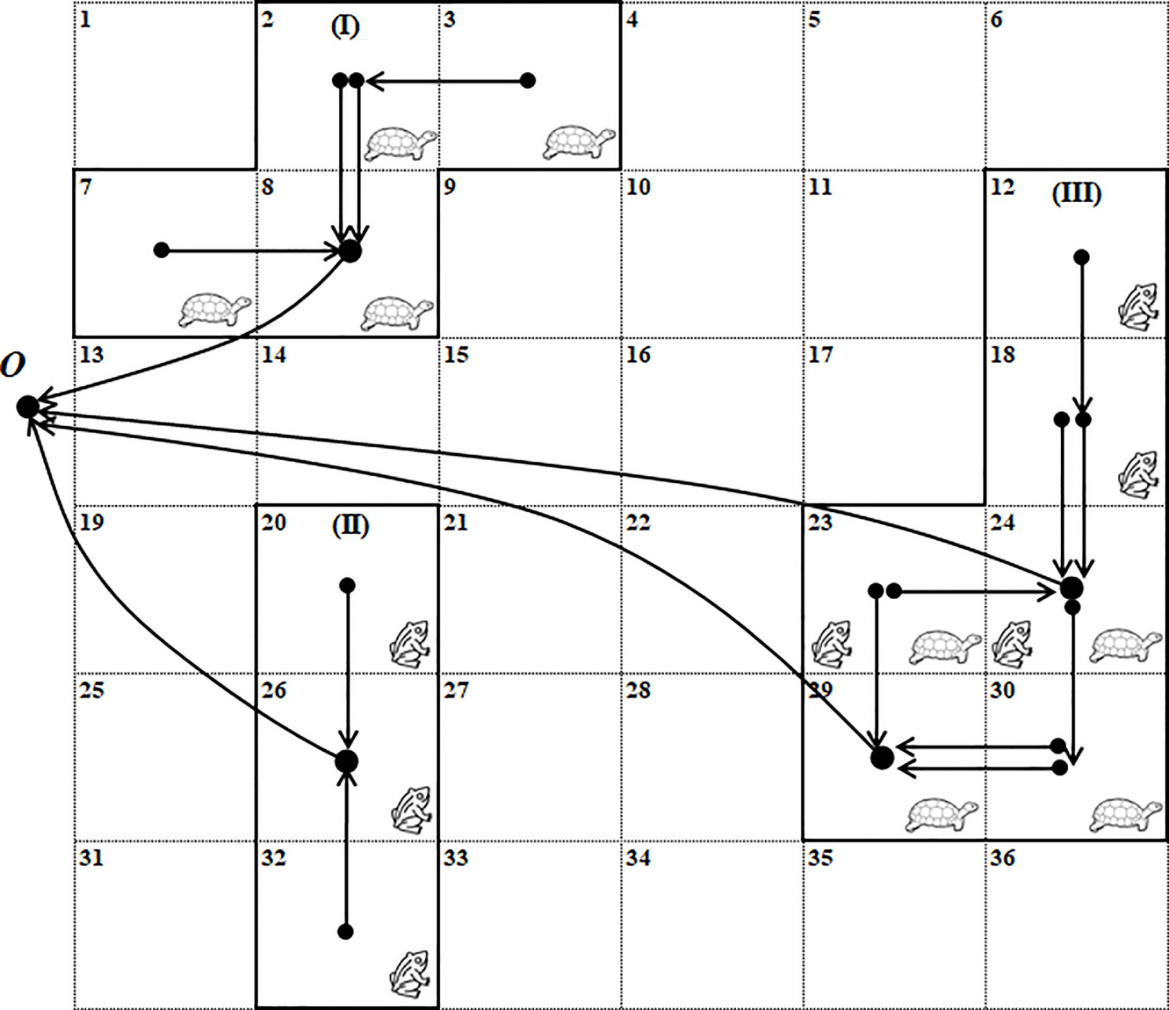
An illustration of three reserves protecting two species (see text for explanations).

We illustrate the above concepts and methods in Fig 1 using a simple case of two species (turtle and frog) and 36 sites (a 6x6 grid). We suppose that both species are ground-bound species, thus both need physical connectivity. In this example, two populations of each species are protected, and the selected sites form three contiguous reserves. Reserve I protects one population of the turtle, and reserve II protects one population of the frog. In reserve III, the sites labeled 23, 24, 29 and 30 protect another population of the turtle, while sites 23 and 24 in conjunction with sites 12 and 18 protect another population of the frog. The selected sites are shown with dots in them, which denote the nodes in the graph. The larger dots (corresponding to sites 8, 26, 24 and 29) indicate the reserve centers. Note that reserve III includes two centers because it is composed of two sub-reserves, each designated to an individual species. The distance between a selected site and the central site of the reserve it belongs can be defined as the number of arcs in the path that connects these two sites. The distance definition is species-specific. For instance, for the turtle, the total distance between the selected sites and the center of reserve I is four, and the total distance in reserve III is also four. For the frog, the distances in reserves II and III are two and four, respectively. Therefore, the total distance (~ compactness) of the entire reserve system is 14 (= 4+4+2+4).

## The model

The notation used in the model, including the indexes, sets, parameters and variables, is given below.

Indices and sets:

*i*, *j*, *k*, *l*, *I*: indices and set of candidate sites;

*s*, *S*: index and set of target species;

*N*_*si*_: set of sites that are adjacent to site *i* for species *s*.

Parameters:

*d*_*ij*_: length of arc (*i*, *j*), measured as the distance between centroids of nodes *i* and *j*;

*r*_*s*_: number of populations to be protected for species *s*;

*e*_*si*_: population size (number of individuals) of species *s* in site *i*;

*p*_*s*_: viable population size of species *s*;

*c*_*i*_: acquisition cost of site *i*;

*B*: conservation budget.

Variables:

*U*_*si*_: binary variable, equals 1 if site *i* is selected for protecting species *s*, 0 otherwise;

*X*_*sijk*_: binary variable, equals 1 if a path connects the nodes associated with site *i* and site *k* through a node associated with site *j* adjacent to site *k*, where sites *i*, *j*, *k* are selected for protection of species *s*, and 0 otherwise; note that *k* can be the auxiliary node *O*. *X*_*siik*_ = 1 means that site *i* is adjacent to site *k*;

*T*_*si*_: binary variable, equals 1 if site *i* is the center of reserve dedicated to species *s*;

*V*_*i*_: binary variable, equals 1 if site *i* is selected (regardless of species type).

The algebraic model is given below.

$$Max\;\sum_{s}\sum_{i}\sum_{j}\sum_{k\in N_{sj}}d_{jk}X_{sijk}$$ (1)

$$\sum_{i\in I}T_{si}=r_{s}\;\mathrm{for}\;\mathrm{all}\;s\in S$$ (2)

$$\sum_{j\in N_{si}}X_{siij}=U_{si}\;\mathrm{for}\;\mathrm{all}\;s\in S,\;i\in I$$ (3)

$$\sum_{j\in N_{sk}}X_{sijk}‑\sum_{l\in N_{sk}}X_{sikl}=0\;\mathrm{for}\;\mathrm{all}\;s\in S,\;i,k\in I,\;k\neq i,\;l\neq i$$ (4)

$$\sum_{j\in N_{sk}}X_{sijk}\leq U_{sk}\;\mathrm{for}\;\mathrm{all}\;s\in S,\;i,k\in I$$ (5)

$$\sum_{j\in I}X_{sijO}=U_{si}\;\mathrm{for}\;\mathrm{all}\;s\in S,\;i\in I$$ (6)

$$X_{sijO}\leq T_{sj}\;\mathrm{for}\;\mathrm{all}\;s\in S,\;i,j\in I$$ (7)

$$T_{si}=X_{siiO}\;\mathrm{for}\;\mathrm{all}\;s\in S,\;\;i\in I$$ (8)

$$\sum_{i\in I}e_{si}X_{sijO}\geq p_{s}*T_{sj}\;\mathrm{for}\;\mathrm{all}\;s\in S,\;j\in I$$ (9)

$$U_{si}\leq V_{i}\;\mathrm{for}\;\mathrm{all}\;s\in S,\;i\in I$$ (10)

$$V_{i}\leq \sum_{s}U_{si}\;\mathrm{for}\;\mathrm{all}\;i\in I$$ (11)

$$\sum_{i\in I}c_{i}V_{i}\leq B$$ (12)

The objective function (1) represents the overall distance between the selected sites in multiple reserves and their respective centers (summed across the species, which is assumed as a measure of overall compactness of the reserve system).

Constraint (2) ensures that for each species *s*, a specified number (*r*_*s*_) of populations, each in a contiguous reserve, is protected. Constraint (3) states that if site *i* is selected to protect species *s* (*U*_*si*_ = 1), then an arc flows from that site into one of its adjacent sites ($\sum_{j\in N_{si}}X_{siij}=1$) that is also designated to species *s*. This constraint also implies that no arc can flow out from unselected sites. Constraint (4) states that if a path originating from site *i* enters into site *k* through a site *j* adjacent to *k*, it must leave site *k* and enter into another adjacent site *l*. If site *i* is selected for species *s* and a path connects it to site *k*, i.e. if $\sum_{j\in N_{sk}}X_{sijk}=1$, constraint (5) requires that site *k* must also be selected for that species, thus *U*_*sk*_ = 1. Constraint (5) also implies that no arc can flow into an unselected site. Constraint (6) ensures that each selected site *i* must be connected to the sink, *O*, by a path. Constraint (7) implies that the last site on such a path must be a central site of a reserve designated to *s* (*T*_*sj*_ = 1). Thus, constraint (6) also implies that when multiple reserves are designated to a species, a selected site belongs to only one of those reserves. Note that a site can belong to different reserves for different species (such as sites 23 and 24 in Fig 1). Constraint (8) ensures that the arcs from center sites flow directly to the sink *O*. Constraint (9) states the minimum viable population restriction, namely if *j* is a central site of a reserve designated to species *s* (*T*_*sj*_ = 1), then the reserve must protect a population of at least *p*_*s*_ individuals. This constraint can be modified to meet alternative conservation targets, such as minimum habitat area or occurrence probability. Constraint (10) states that if a site is part of a reserve designated to any species, it must be selected in the first place, and constraint (11) requires that any selected site must serve for the protection of at least one species. Finally, constraint (12) is the budget constraint. Note that variables *T*_*si*_ and *U*_*si*_ are redundant and can be replaced with *X*_*siiO*_ and $\sum_{j\in N_{si}}X_{siij}$, respectively. However, including these variables makes the model easier to understand. Moreover, according to our computational experience, this helps improve the model’s computational efficiency (possibly due to a change in the progress of the branch-and-bound procedure. Similar observations were reported in other studies as well, e.g. [33]).

All parameters in the model except the acquisition cost (*c*_*i*_) can be determined or estimated based on biological requirements. The minimum viable population size for individual species (*p*_*s*_) can be estimated using population viability analysis [34]. Species distribution across the candidate sites (*e*_*si*_), whether in the form of presence-absence, occurrence probability, individual abundance or other forms, may be determined based on species distribution models [35]. The number of populations to be protected (*r*_*s*_) should be determined by the conservation planner based on the species’ distribution and its biological status such as threatened or endangered. The parameters *q*_*si*_ can be the amounts of food and shelter provided by individual sites or species-specific habitat quality indicators such as species demographics and distributional patterns [36]. It is also reasonable to suppose that *q*_*si*_ is dependent on the number of site *i*’s neighbors (immediately adjacent sites) that are selected and which species are protected by those sites. Writing this relationship mathematically would require defining additional binary variables. For simplicity, here we assumed that *q*_*si*_ is a fixed value. The site acquisition costs (*c*_*i*_) are typically estimated based on land prices or assessed land values [37].

### Computational efficiency

We tested the computational efficiency of the model (1)-(12) for reserve site selection problems using synthetically generated data sets involving square grid partitions. We started with a 100-site problem with one species to protect, and gradually increased the problem size considering up to 1000 sites and 10 species. To eliminate the impact of input data on computational efficiency, for each problem we generated 30 different data sets, where each run assumed a different species distribution, habitat quality for individual sites, and site costs. We randomly generated the species distribution data and the habitat quality data from uniform [0, 10] and uniform [0, 5] distributions, respectively, in such a way that a site’s habitat quality for a species is positively related to the species’ presence and abundance in that site. Likewise, we randomly generated the site costs using a uniform [1, 10] distribution. In all runs, we imposed that one population of each species consisting of at least 40 individuals must be protected (i.e., *r*_*s*_ = 1 in constraint 2 and *p*_*s*_ = 40 in constraint 9). We set the conservation budgets availability as *B* = 20, 50, and 100 for the scenarios targeting one, five, and ten species, respectively. We solved the model using GAMS 23.9/GUROBI 5.0 on a personal computer with a CPU of 2.8 gigahertz and a RAM of 8 gigabytes. To save processing time (CPU) in the test runs, we set the relative optimality criterion at one percent (i.e. optcr = 0.01 in GAMS/GUROBI). We specified that the length of any path that connects site *i* to its corresponding central site *j* cannot be greater than or equal to a specified distance threshold (which was set as four). This is because we minimize the total distance between the selected sites and the reserve centers; therefore long, meandering reserves are not likely to be generated. We also limited the processing time to one hour for each model run and limited the total run time to two hours (that is, the solver was forced to terminate within the two-hour time limit regardless of whether 30 runs are completed or not). In Table 1, we report the models’ sizes and average CPU times needed to solve the problem only for the completed runs.

**Table 1:** Model sizes and CPU times needed to solve the model in the computational efficiency test.

Number of sites	Number of species		
	1	5	10
100 = 10*10	31; 46 ^a^	152; 231	305; 462
	0.6	58.0	471.0
200 = 10*20	121; 189	604; 942	1,210; 1,884
	2.3	584.1	643.4
400 = 20*20	482; 769	2,408; 3,844	4,820; 7,688
	12.0	225.6	750.9
800 = 25*32	1,924; 3,111	9,617; 15,553	19,233; 31,105
	62.6	^b^	^b^
1000 = 20*50	3,005; 4,863	15,021; 24,311	^b^
	96.8	^b^	

^a^ In the table, the number before a semicolon is the number of equations and the number after a semicolon is the number of variables involved in the model (*1000).

^b^ Memory limit* was hit when solving the problem or generating the MIP model. [*GAMS/GUROBI requires an estimated amount of workspace to generate the model and save temporary files created during the solution process. This is based on the model statistics (number of variables, equations, density, etc.). The estimated memory may become insufficient when solving large models. See, https://www.gams.com/latest/docs/S_GUROBI.html.]

The number of equations and the number of variables included in the model increased both as more candidate sites and more species are involved. Our experiments with the model showed that the time needed to find the optimal solution increased as more candidate sites are considered. For instance, when the number of candidate sites was specified as 100 and one species was to be protected, the model could be solved within only 0.6 seconds. The solution time gradually increased to 96.8 seconds as the number of sites was increased to 1000. A similar pattern was observed when more species were included in the model. This is expected because including more candidate sites and more species results in a larger MIP model which typically requires more processing time to solve. However, this was not always the case. When five species were included and the number of candidate sites was 200, only 16 runs were completed within the two-hour time limit. The average processing time for each run was 584 seconds. The latter decreased to 226 seconds when the number of candidate sites was increased to 400 (all 30 runs were completed within two hours). When the number of candidate sites was 800 and 1000, and five or ten species were included, GUROBI ran out of memory.

### An empirical application

The United States Army installations use approximately 12.2 million acres (49.4 thousand km^2^) of land in the continental United States. By securing ecosystems from land conversion and harboring many threatened or endangered species, these military installations have significant potential to contribute to regional and global biological conservation [38]. Among these installations, Fort Stewart in Georgia has the greatest number of federally and state-listed amphibian and reptile species. One of those species is Gopher Tortoise (*Gopherus polyphemus*), hereafter referred to as GT, which is native to the southeastern United States. GTs are a keystone species currently listed as state-threatened in Georgia and a candidate to be listed as federally-threatened. Much of the GT natural habitats (sandy soils covered with longleaf pine forests that provide a suitable canopy for herbaceous plant growth) have been lost or reduced dramatically due to the conversion of those lands to agriculture and urban development. To cope with the ongoing habitat loss, some GT populations are being relocated from the surrounding areas into Fort Stewart. Another important species in the region is Gopher Frog (*Lithobathes capito*), hereafter referred to as GF. The GFs are under review by the U.S. Fish and Wildlife Service and have a vulnerable status according to NatureServe [39]. GFs live in upland areas surrounding ponds and other small water bodies which are essential for their reproduction. When open-air conditions become unfavorable, the GFs seek shelter in nearby GT burrows to avoid fire, extreme heat, cold, and dry weather. There are numerous ponds, the majority of which are seasonal, scattered across Fort Stewart. The purpose of our analysis here is to determine the most suitable habitat areas that can support a targeted GT population to be relocated from outside the boundaries of the installation while also providing adequate protection to cohabiting GFs. Such areas are called Conservation Management Areas (CMA). Technically, these areas are not considered as “conservation reserves” because they are military lands under all circumstances. Yet, land managers are required to implement appropriate management practices, such as prescribed burns and maintaining a suitable forest canopy and understory vegetation. Therefore, protecting habitat areas does not necessarily mean taking those lands out of the military uses. Previous work focused on creating compact reserves [40]. Since GTs are ground-bound species, the selected CMAs must be both compact and contiguous. Simultaneous consideration of GFs would give preference to areas that have ponds within and around them so that GFs can have access to those ponds while being protected by the GT burrows when needed.

We overlaid the installation area by a square grid partition that includes 30 rows and 55 columns where each cell in the partition has an area of 1 km^2^. We considered each cell as a spatial land unit that can be selected or left out when configuring the boundaries of CMAs. The relevant data were obtained from the installation’s ecosystem/landscape managers (for details, see [40]). In the data, the carrying capacity of individual cells ranges between 0 to 601 GTs per cell, and the number of ponds in each cell ranges between 0 to 13. GFs can travel distances up to one mile. Therefore, for simplicity, we assumed that GFs cohabiting the selected GT cells can have access to the ponds only in those cells and in their immediate neighbors.

We applied the model described by (1)-(9) only to GT because reserve contiguity and compactness are required only for GTs. In addition to constraint (9), which specifies the population requirement at individual CMA level (where we set *p*_*s*_ = 1000), we also imposed a similar requirement for the entire CMA system as below:

$$\sum_{i}qt_{i}*U_{i}\geq gt$$ (13)

where *qt*_*i*_ is the carrying capacity of cell *i* for GTs, and *gt* denotes the total carrying capacity of selected cells for GTs, which is the size of the relocated GT population. In this application, we specified *gt* = 5000 individuals.

Since the area considered for conservation is part of a military installation, no rental or purchase cost (budget) is involved. Instead, in constraint (12), $\sum_{i\in I}U_{i}\leq B$, we set *B* as the total number of selected GT cells. This proxy budget specification is meaningful because the number of selected GT cells affects the cost of canopy and vegetation management. We specified the right-hand side of (12) as *B* = 30.

For GF, we modified constraints (10) and (11) as follows:

$$V_{i}\leq U_{i}\;\mathrm{for}\;\mathrm{all}\;i$$ (14)

$$\sum_{j\in N_{i}}p_{j}\geq f*V_{i}\;\mathrm{for}\;\mathrm{all}\;i$$ (15)

$$\sum_{i}V_{i}\geq 0.5\;*\sum_{i}U_{i}$$ (16)

Constraint (14) ensures that the cells designated for GF are among the selected GT cells (but not vice versa). Constraint (15) states that the number of ponds in and around each designated GF cell must be at least *f*, which represents the abundance of ponds in the immediate neighborhood of each cell (including the cell itself). In our data set, the average number of ponds across all cells is approximately 1.7, thus the average number of ponds in and around each cell is 9*1.7 = 15.3. We imposed a somewhat higher abundance level than this average, specifically *f* = 20. Constraint (16) ensures that at least half of the selected GT cells must be designated GF cells. Some of the assumptions described above are specified arbitrarily for demonstration purposes rather than representing true ecological requirements.

We generated alternative solutions first considering the two cohabiting species together, and then considering only the keystone species (GT). The purpose is to investigate how the configuration and locations of the protected areas are impacted when the spatial interaction between the two species is included as a driving factor in site selection, which is the primary motivation of the present study. We also determined the optimal configuration of one large and two small CMAs in each case to investigate the variations of compactness and the habitat qualities of the designed CMAs.

**Fig 2:**
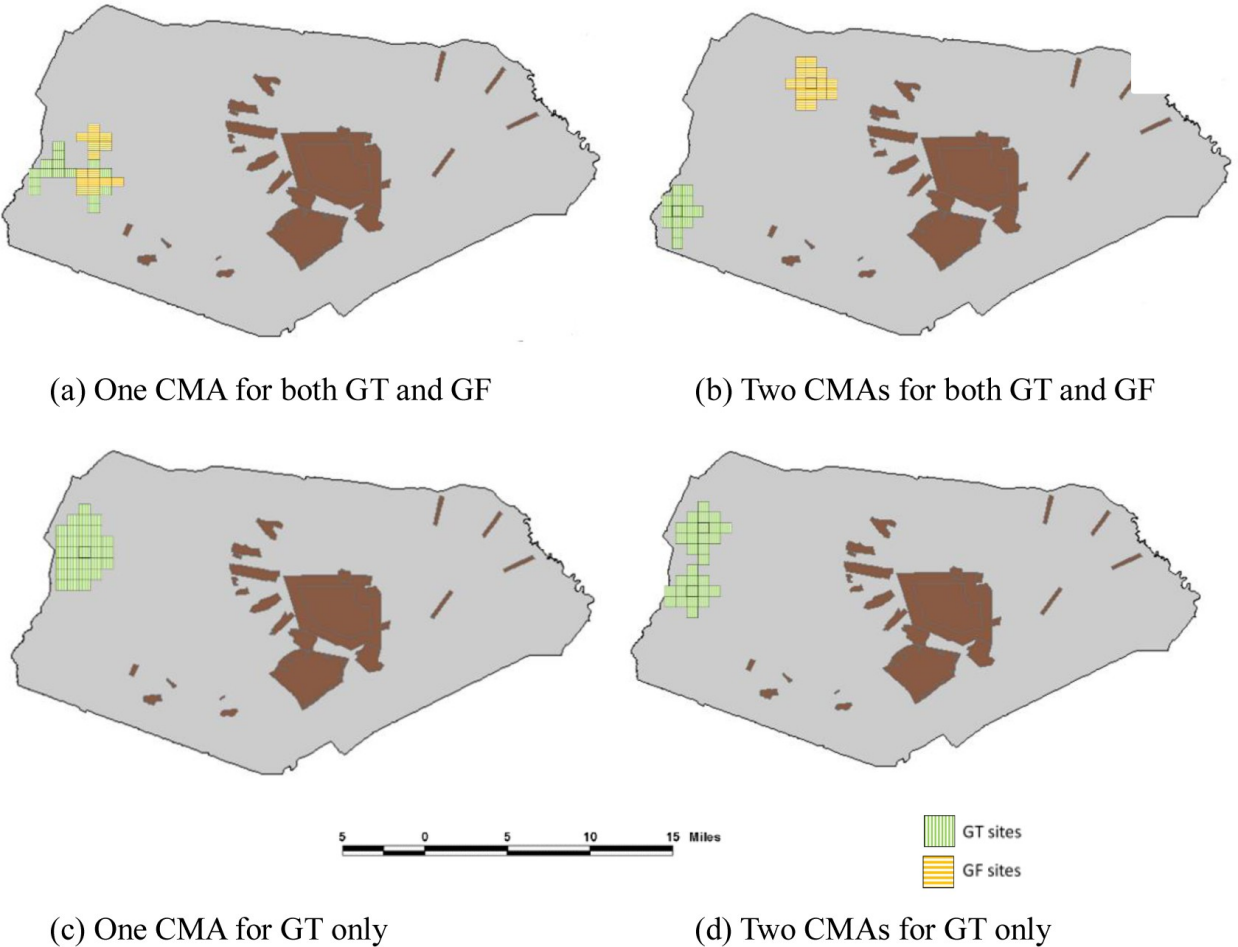
Location and layouts of selected CMAs in the empirical application. Green cells are the GT cells, orange cells are the GT cells that are also designated GF cells. The brown colored areas are the intensively used military training ranges.

Fig 2 displays the configurations and locations of the selected CMAs. Table 2 presents a summary of the compactness and habitat quality metrics calculated in each case. Several noteworthy conclusions can be made based on the model results. First, when a single large CMA was designed to protect both GT and GF, the total distance between the selected cells and the central cell was found as 105 (Table 2, the total distance panel; we note that the total distance was calculated considering only the cells designated to GT because spatial contiguity and compactness are required only for GT). This distance decreased substantially to 53 when two CMAs were designed. The configurations and locations of the selected CMAs are shown in panels (a) and (b) of Fig 2. The same pattern is observed when only GT is targeted for protection and one and two CMAs are configured, where the total distances were 79 and 52, respectively. This is an expected and intuitive result because configuring more than one CMA reduces the size of each CMA (although the total area remains the same) and makes the selected cells closer to the central cells. Second, compared with the solutions for GT only, targeting both GT and GF alters the location and configuration of the CMAs dramatically. When only GT is considered and one large CMA is to be configured, the selected cells would be located again in the western part of the installation, but now they are clustered together and form a substantially more compact spatial configuration (see panels a and c). This result is based on the fact that this area provides the most suitable habitat areas for GT. The inclusion of GF in site selection compromises spatial compactness of the CMA due to the selection of sites in favor of GF. The changes in site selection and the spatial layout of the reserve system are much more pronounced when two CMAs are configured and both species are considered simultaneously. In this case, one of the CMAs is moved eastward and is quite distant from the other CMA (see Fig 2, panels b and d). Third, the total habitat quality of the conservation management areas increased when two smaller CMAs were configured compared to one single large CMA. For instance, when GT and GF are targeted together, the total habitat quality of the GT and GF reserves increased from 79.5 (= 47.8+31.7) to 83.8 (= 53.0+30.8; the added figures represent the total standardized habitat quality indexes for GT and GF, respectively). The same pattern is observed (to a much lesser extent, though) when only GT is targeted for protection. These results indicate that two CMAs may be preferable than one CMA both in terms of compactness and habitat quality of the conservation areas.

**Table 2:** The compactness and habitat quality metrics of designed CMAs in the empirical application.

	Total distance		Total quality	
	One CMA	Two CMAs	One CMA	Two CMAs
GT and GF	105	53	47.8 (GT)	53.0 (GT)
			31.7 (GF)	30.8 (GF)
GT only	79	52	47.2	47.7

We defined the total quality as the sum of standardized habitat qualities of the selected cells considering the two species separately. For GTs, the habitat quality of a cell is defined as the cell’s carrying capacity. For GFs, it is defined based on the cell’s carrying capacity for GT as well as the number of ponds within the cell and its adjacent cells.

In general, solving medium and large-scale integer programming models to ‘exact optimality’ (i.e. with zero suboptimality tolerance) can be computationally challenging because of long processing times. If the allowed solution time is not enough, an ‘optimum’ solution even with a reasonably small suboptimality allowance may not be obtained by the solver. Reducing the model size without sacrificing from optimality is often necessary to avoid this. In this empirical application, we excluded the poor-quality cells and small isolated areas (detached from the remaining eligible cells) to reduce the model size to a computationally manageable level. This left 890 cells to be included in the model. It took about 15 minutes of processing time to solve the model for one CMA when targeting both GT and GF, and 5 minutes when targeting only GT. The solution time was reduced to 30 seconds for two CMAs in both targeting scenarios.

## Conclusions

Optimizing conservation planning for multiple cohabiting species must take into account the spatial needs of those species and spatial attributes of the individual candidate sites to increase the effectiveness and efficiency of the reserves. Incorporating spatial criteria, especially contiguity and compactness, in an optimization framework is a challenging problem because of modeling and computational complexities. Existing mixed integer programming (MIP) models presented in the conservation biology literature are either inconvenient to use or not species-specific. In this paper, we have presented a linear MIP model for determining the optimal design of a nature reserve system for multiple cohabiting species with joint consideration of contiguity and compactness as the spatial criteria in site selection. To promote compactness, the model minimizes the total distance between selected sites and central sites, both determined by the model simultaneously, across all reserves. It uses graph theoretic concepts (paths) to ensure reserve contiguity for individual species that need physical connectivity. The uniqueness of the model lies in the fact that it measures the distance between the selected sites and the central sites along the paths composed of only selected sites. Using the model, we determined optimal conservation management areas for two cohabiting species, Gopher Tortoise (*Gopherus polyphemus*) and Gopher Frog (*Lithobathes capito*), in a military installation (Fort Stewart in Georgia, USA). Our method is quite general and applicable to conservation planning problems where site selection decisions must take into account similar spatial criteria.

In the empirical application presented here, we allowed one or two reserves, each satisfying the contiguity and compactness criteria and together meet the population coverage (conservation) constraint. Allowing multiple reserves would promote small protected areas, possibly distant from each other, which may not be a desirable situation in most empirical cases. Such undesirable outcomes can be avoided (or controlled) by imposing an upper bound on the number of such reserves that can be generated by the model. This can be done by introducing a new binary variable *Y*_*i*_ and adding to the model the following two constraints, *T*_*si*_≤*Y*_*i*_ for all *s* and *i*, and $\sum_{i}Y_{i}\leq tr$. Here *Y*_*i*_ indicates whether site *i* is a reserve center (*Y*_*i*_ = 1) or not (*Y*_*i*_ = 0). The first of these two constraints implies that if site *i* is a center (*T*_*is*_ = 1), then there is a reserve centered at site *i*, i.e., *Y*_*i*_ = 1. The second constraint implies that the total number of such reserves cannot exceed *tr*, a positive integer specified by the conservation planner. One can also incorporate the sum of distances between those reserve centers as an additional term in the objective function to discourage the formation of a reserve system with highly scattered reserves.

Our computational experience with the model shows that medium to large-scale optimum reserve selection problems for multiple cohabiting species can be solved relatively easily, but very large instances of such problems can be difficult to solve to optimality in reasonable processing time. However, the ongoing progress in computational power and optimization software development has been dramatic in the past two decades. If the progress continues at this pace, the popularity of optimization methods in conservation planning would be much higher, especially in a more resource-constrained future.

## Supporting information

S1 Data GAMS code used in the computational efficiency test. (GMS)
